# Glycan recognition in globally dominant human rotaviruses

**DOI:** 10.1038/s41467-018-05098-4

**Published:** 2018-07-06

**Authors:** Liya Hu, Banumathi Sankaran, Daniel R. Laucirica, Ketki Patil, Wilhelm Salmen, Allan Chris M Ferreon, Phoebe S. Tsoi, Yi Lasanajak, David F. Smith, Sasirekha Ramani, Robert L. Atmar, Mary K. Estes, Josephine C. Ferreon, B. V. Venkataram Prasad

**Affiliations:** 1 Verna and Marrs McLean Department of Biochemistry and Molecular Biology, Baylor College of Medicine, Houston, TX 77030 USA; 2 Molecular Biophysics and Integrated Bioimaging, Berkeley Center for Structural Biology, Lawrence Berkeley National Laboratory, Berkeley, CA 94720 USA; 3 Department of Molecular Virology and Microbiology, Baylor College of Medicine, Houston, TX 77030 USA; 4 Department of Pharmacology, Baylor College of Medicine, Houston, TX 77030 USA; 5 Department of Biochemistry and the Emory Comprehensive Glycomics Core, Emory University School of Medicine, Atlanta, GA 30322 USA; 6 Department of Medicine, Baylor College of Medicine, Houston, TX 77030 USA

## Abstract

Rotaviruses (RVs) cause life-threatening diarrhea in infants and children worldwide. Recent biochemical and epidemiological studies underscore the importance of histo-blood group antigens (HBGA) as both cell attachment and susceptibility factors for the globally dominant P[4], P[6], and P[8] genotypes of human RVs. How these genotypes interact with HBGA is not known. Here, our crystal structures of P[4] and a neonate-specific P[6] VP8*s alone and in complex with H-type I HBGA reveal a unique glycan binding site that is conserved in the globally dominant genotypes and allows for the binding of ABH HBGAs, consistent with their prevalence. Remarkably, the VP8* of P[6] RVs isolated from neonates displays subtle structural changes in this binding site that may restrict its ability to bind branched glycans. This provides a structural basis for the age-restricted tropism of some P[6] RVs as developmentally regulated unbranched glycans are more abundant in the neonatal gut.

## Abstract (web summary)

Human rotaviruses (RV) bind to histo-blood group antigens (HBGA) for attachment, but how different viral genotypes interact with HBGA isn’t known. Here, Hu *et al*. report crystal structures of a prevalent and a neonate-specific RV in complex with HBGA and provide insights into glycan recognition and age-restricted tropism of RVs.

## Introduction

Rotaviruses (RVs) cause acute gastroenteritis in infants and children under the age of 5 years resulting in an estimated 215,000 deaths worldwide annually^1^. RV is a non-enveloped double-stranded RNA (dsRNA) virus with three concentric capsid layers encapsidating eleven dsRNA segments. The outermost layer contains the glycoprotein VP7 and the protease-sensitive spike protein VP4, which define the classification of RVs into G and P genotypes, respectively^2^. RVs display enormous genetic diversity, with 35G and 50P genotypes identified so far^3,4^. Based on phylogenetic analysis, the P genotypes have further been classified into five genogroups^5^. Among the P genotypes, P[4], P[6], and P[8] represent the most commonly circulating genotypes associated with most human RV infections worldwide^6–9^. P[8] is also the VP4 genotype of the two live attenuated RV vaccines used globally^10^. These genotypes, along with P[19] that mainly infects pigs, are classified into the P[II] genogroup^5^ (Supplementary Fig. 1). Compared to P[4] and P[8] RVs, P[6] RVs have a restricted global prevalence and are more commonly found in sub-Saharan Africa and southeast Asia including India^9,11,12^. Further, P[6] infections in neonates have been described in many countries^13,14^. A P[6] RV strain (RV3) that showed such age-restricted tropism and caused asymptomatic infection in neonates is being developed as a vaccine candidate in Australia^15^. It has been suggested that the VP4 gene segment is likely the principal determinant of the geographic and age-restricted spread of P[6] RVs among human populations^9,16^.

The initial attachment of viruses to cellular glycan receptors is a critical determinant of host specificity, tissue tropism, and zoonotic transmission^17^. RVs use the VP8* domain of the spike protein VP4 to recognize specific host glycans in a genotype-dependent manner^18^. It has been shown that while the majority of animal RVs (ARVs) recognize sialoglycans^19–22^, some ARVs and human RVs (HRVs) specifically bind to polymorphic histo-blood group antigens (HBGAs) that are present on gastric epithelial cells and in mucosal secretions^23–28^. HBGAs are blood-type determinants that represent terminal structures in the glycan chains^18,29^. The expression of HBGAs is genetically determined and is based on an individual’s ABO, secretor and Lewis status. Recent epidemiological studies indicate that infection by RVs strongly correlates with the secretor status of the individual, suggesting that the HBGAs are susceptibility factors for HRVs as well as cell attachment factors^30–34^.

**Fig. 1:**
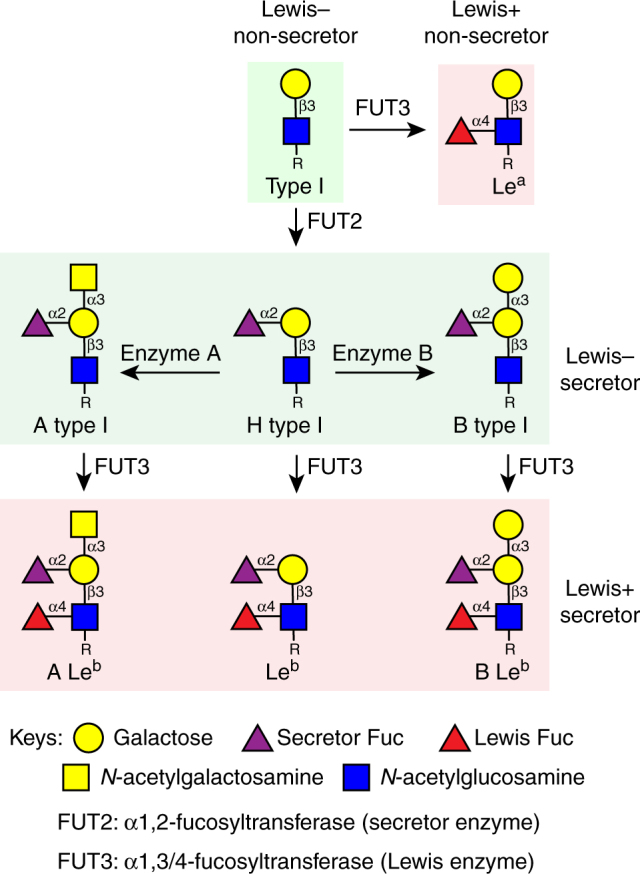
Schematic diagram of type I HBGA synthesis shows the structures of HBGAs in secretor and non-secretor individuals

HBGAs are synthesized by sequential addition of a carbohydrate moiety to the precursor disaccharide β-galactose-*N*-acetyl-glucosamine (β-Gal-GlcNAc) with a β1,**3** (type I) or a β1,**4** (type II) linkage resulting in distinct ABH and Lewis HBGAs^29^. In secretor-positive individuals with a functional α-1, 2 fucosyltransferase (FUT2), an α-fucose (SeFuc) is added to the β-Gal residue of the precursor resulting in H-type HBGA; subsequent addition of *N*-acetylgalactosamine (GalNAc) by enzyme A or α-galactose (α-Gal) by enzyme B to the precursor β-Gal results in A or B-type HBGAs, respectively (Fig. 1). Further modification of ABH HBGAs can occur by α-1, 3 fucosyltransferase (FUT3); adding an α-Fuc (LeFuc) to the precursor GlcNAc leads to secretor-positive Lewis (Se + Le+) HBGAs (ALe^b^, BLe^b^, and Le^b^). In the case of non-secretor individuals, only FUT3 is functional and LeFuc is added to the precursor GlcNAc, resulting in the secretor-negative Lewis (Se−Le+) HBGAs. The expression of HBGAs is developmentally regulated. In neonates, precursor disaccharides are expressed and the addition of branches and terminal carbohydrates is regulated in a tissue-specific manner during development^26,35^.

X-ray crystallographic studies of VP8* in complex with HBGAs have provided critical insight into RV host–pathogen interactions^23,24,36^. Previous crystallographic studies revealed that the VP8* of the P[14] HRV binds to A-type HBGA at a site that overlaps with the sialic acid binding site in ARVs, providing insight into inter-species transmission of an animal-origin virus to the human host^23^. Similarly, a neonate-specific bovine-human reassortant P[11] HRV VP8* recognizes type I and type II precursor glycans that are expressed in the neonatal gut and human milk, while its bovine counterpart only binds to type II glycans^24^, consistent with their abundance in bovine milk. However, with the exception of these initial structural studies on less common HRV strains, our understanding of genotype-dependent glycan recognition in the globally dominant HRV strains is still limited and there are as yet no structural studies describing how these strains recognize glycans.

The VP8*s of P[II] genogroup RVs, which include the globally dominant P[4], P[6], and P[8] strains and the rare P[19] genotype, are known to recognize H-type I HBGA as well as mucin cores^27,37^. Using saturation transfer difference (STD) NMR and crystallography, it has been recently shown that the P[19] VP8* binds to type I glycans at a novel glycan binding site that is distinct from the previously characterized A-type or precursor binding sites in RVs of P[14] and P[11] genotypes^27,36^. STD NMR, glycan binding, and infectivity assays have shown that P[4] and P[6] HRVs, but not P[8], bind to A-type HBGA^28^. However, although the VP8* structures of P[4] HRV (DS-1-like) and P[8] HRV (Wa-like) in isolation have been determined^20,38^, it is not known how they bind to cellular glycans due to the lack of their structures in complex with any glycans. Our previous glycan array studies with 247 human milk glycans (HMGs) revealed that VP8* of the neonate-specific P[6] RV (vaccine strain RV3) recognizes H-type I HBGA and a type I glycan with a sequence of Galβ1,**3**-GlcNAcβ1,3-Galβ1,4-Glc (also known as lacto-*N*-tetraose, LNT)^39,40^. However, no structures of neonate-specific P[6] HRVs, in isolation or in complex with glycan have been described to date.

Here, using X-ray crystallography, we provide the structural basis of the glycan recognition by the globally prevalent HRVs of P[4] and P[6] genotypes. Our studies show that VP8* of these genotypes engages the type I precursor glycan (Galβ1,**3**-GlcNAc) at a unique and conserved binding site that is present in all P[4], P[6] and P[8] HRVs, and the subtle sequence and structural changes lead to an altered glycan configuration at the reducing end in the neonate-specific P[6] RV, that may play a key role in the age-restricted tropism of some P[6] strains.

## Results

### Crystal structure of a neonate-specific VP8* P[6] HRV

**Fig. 2:**
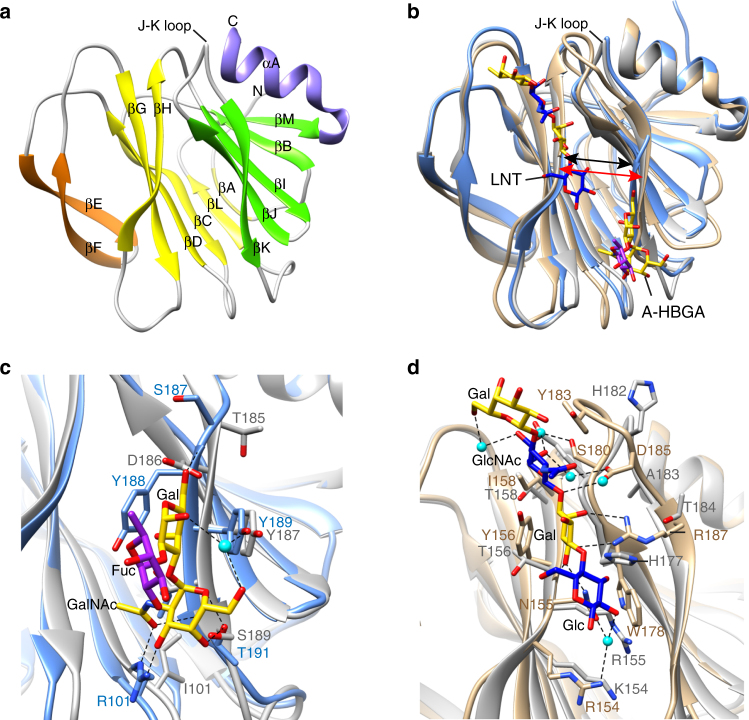
VP8* structure of a neonatal human rotavirus strain P[6] RV3 and structural comparison with VP8*s of other HRVs in complex with HBGAs. **a** Ribbon representation of the P[6] RV3 VP8* structure displays a galectin-like fold with the two twisted β-sheets in yellow and green, respectively. The β-hairpin and the C-terminal α-helix and are colored in orange and purple, respectively. **b** Structural superposition of P[6] VP8* (gray) with HRV P[14] VP8* (light blue) in complex with A-type HBGA (PDB ID: 4DRV) and HRV P[11] VP8* (tan) in complex with LNT (PDB ID: 4YFZ). The width of the cleft between the β-sheets in P[6] and P[11] VP8*s is wider than that in P[14] VP8*, as indicated by red and black arrows, respectively. The glycans bound on VP8* are represented with sticks. Close-up views of the A-type HBGA (**c**) and LNT (**d**) binding sites show the amino acid changes in P[6] VP8* disallow the glycan binding at these sites. The interacting residues are shown in stick model and labeled. The proteins are colored as in **b**

As noted above, the structure of the neonate-specific P[6] VP8* has not been determined previously. To examine whether the VP8* structure of P[6] differs from that of other HRV genotypes including P[4], P[8], P[11], and P[14], and whether it can bind glycans as observed in P[11] and P[14], we first determined the crystal structure of VP8* of a neonate-specific P[6] HRV strain RV3 at 2.0 Å resolution (Fig. 2a, Table 1). Similar to other VP8* structures, the P[6] VP8* has a β-sandwich galectin fold with a distinct cleft between the two twisted β-sheets. The cleft in the P[6] VP8* is noticeably wider compared to that observed in the VP8* of P[14] HRV, but similar to that observed in the VP8* of other HRV genotypes including P[4], P[8], and P[11] (Fig. 2b). Comparative analysis with the glycan bound structures of P[14] and P[11] VP8*s shows how sequence variations in the P[6] VP8* leads to the loss of A-type HBGA binding observed with P[14] VP8* (Fig. 2c) and how interaction with type I glycans in P[6] VP8* is unlikely to be similar to that observed in P[11] VP8* (Fig. 2d). The sidechain of R101 in P[14], which makes a strong hydrogen bond interaction through its side chain with A-type HBGA, is substituted by isoleucine in P[6] which cannot make such an interaction; the hydrophobic residue Y188, which is crucial for stabilizing A-type glycan in P[14] VP8*, is replaced by a hydrophilic amino acid D186 in the P[6] VP8* (Fig. 2c). Both P[6] and P[11] VP8*s bind type I glycans such as LNT and H-type I on a shotgun milk glycan array^39,40^, and the LNT binding site in the P[11] VP8* is structurally characterized^24^. There are marked differences in the region in P[6] VP8* structure that corresponds to the LNT binding site in the P[11] VP8* (Fig. 2d). The residue R187 involved in direct hydrogen bonds with Gal of LNT in P[11] VP8* is replaced by T184 in P[6] VP8*. The residues Y156 and W178, which provide hydrophobic interaction with LNT in P[11] VP8*, are changed to T156 and H177, respectively. The J-K loop that interacts with LNT in P[11] VP8* projects away in the P[6]VP8* structure. These observations indicate that the neonate-specific P[6] VP8* cannot bind LNT at the same site as P[11] VP8* and likely has a distinct site for type I glycan binding.

**Table 1:** Crystallographic statistics of P[6] RV3 VP8* and P[4] Indian VP8* structures

	P[6] RV3 VP8*	P[6] RV3 VP8*/LNFP1	P[4] Indian VP8*	P[4] Indian VP8*/ LNFP1
PDB ID	5VX8	5VX9	5VX4	5VX5
Data collection				
Space group	P 1	P 2_1_ 2_1_ 2_1_	P 2_1_	P 2_1_ 2_1_ 2_1_
Cell dimensions				
* a*, *b*, *c* (Å)	27.79, 43.10, 65.74	36.51, 42.63, 95.07	34.33, 56.59 37.31	33.79, 50.24, 110.82
* α*, *β*, *γ* (°)	94.86, 99.40, 90.22	90, 90, 90	90, 93.52, 90	90, 90, 90
Wavelength (Å)	0.9774	1.0	0.9774	0.9774
Resolution (Å)	27.41–2.00 (2.11–2.00)	36.51–1.82 (1.92–1.82)	31.11–1.55 (1.61–1.55)	28.04–1.29 (1.33–1.29)
*R*_merge_ (%)	7.1 (10.2)	9.1 (18.3)	4.7 (22.8)	5.6 (26.9)
Number of molecules in the asymmetric unit	2	1	1	1
ompleteness (%)	90.5 (83.4)	99.1 (98.9)	94.46 (92.31)	93.41 (85.74)
Redundancy	2.1 (2.1)	4.7 (4.8)	2.0 (2.0)	2.0 (1.9)
Refinement				
Resolution (Å)	27.41–2.00	34.08–1.82	31.11–1.55	28.04–1.29
No. of reflections	33,614	25,104	38,653	85,379
* R*_work_/*R*_free_	21.01/25.79	14.32/19.94	17.12/22.10	15.33/16.76
Average B factor (Å^2^)				
Protein	13.75	11.60	13.18	8.69
Ligand	30.93 (SO4)	9.90 (LNFP1) 20.30 (SO4)	12.44 (Tris)	15.09 (LNFP1)
Water	22.38	23.82	25.38	24.60
R.M.S. deviations				
Bond length (Å)	0.005	0.007	0.007	0.006
Bond angle (°)	0.712	1.094	1.04	1.09

### Neonate-specific P[6] HRV binds to type I glycan uniquely

**Fig. 3:**
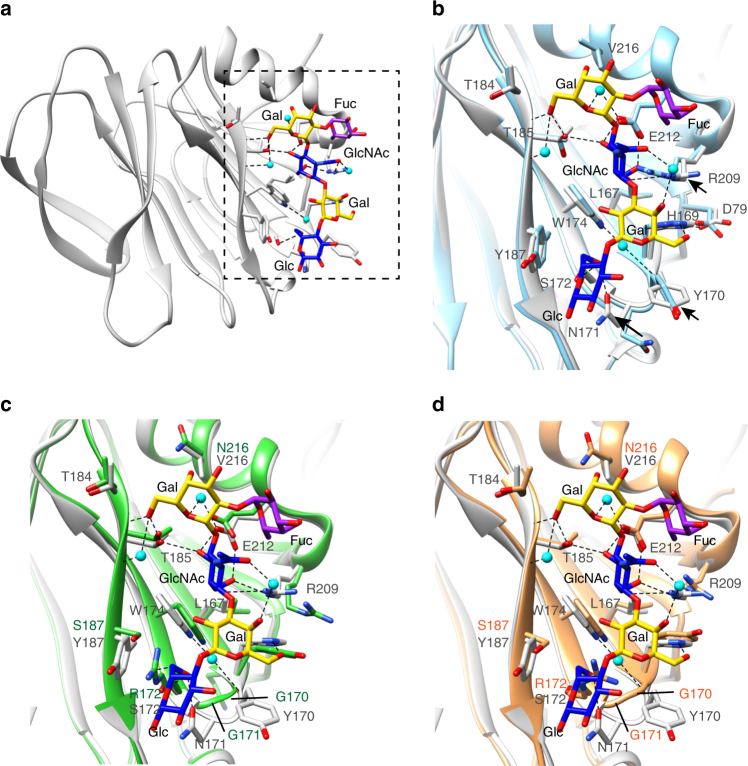
Structure of P[6] RV3 VP8* in complex with H type I pentasaccharide. **a** Structure of P[6] RV3 VP8* is shown in gray ribbon, with the bound glycan in stick model. The glycan residues are labeled. **b** Superimposition of P[6] VP8* apo structure (cyan) and the liganded P[6] VP8* (gray) shows the structural change of VP8* upon binding to H type I. The changes of sidechain orientations are indicated by black arrows. **c** Structural alignment of the structure of P[6] RV3 VP8* (gray) in complex with H type I and P[4] DS-1 VP8* (green) apo structure (PDB ID: 2AEN) shows how the Gal-GlcNAc moieties can bind to the conserved the amino acids in P[4] VP8*, and how the changes in P[4] DS-1 lead to loss of interaction with the Glc at the reducing end of H-type I glycan. **d** Structural alignment of the structure of P[6] RV3 VP8* (gray) in complex with H type I and P[8] Wa VP8* (orange) apo structure (PDB ID: 2DWR). The structures are shown as in **c**

Our previous glycan array studies have shown that VP8* of neonate-specific P[6] RV3 binds to type I glycans containing a common sequence of Galβ1,**3**-GlcNAcβ1,3-Galβ1,4-Glc, with and without a α1,2-linked SeFuc to Gal at the non-reducing end, which represents H-type I and precursor HBGA, respectively. To understand how the VP8* of neonate-specific P[6] HRV recognizes type I glycans, we determined the structure of P[6] VP8* with H-type I pentasaccharide (also known as Lacto-*N*-fucopentaose I or LNFP1) at the resolution of 1.8 Å. Distinct from the HBGA binding sites in P[11] and P[14] HRVs or the Sia-binding site in ARVs, the H-type I glycan binding site consists of one of the β-sheets and the C-terminal α-helix of P[6] VP8* (Fig. 3a, Supplementary Fig. 2A and Table 1). The residues L167, W174, T184, T185, R209, and E212 are involved either in hydrophobic interactions or in a network of direct or water-mediated hydrogen bond interactions with type I precursor Galβ1,3-GlcNAc at the non-reducing end (Fig. 3b and Supplementary Fig. 3A). The Gal4 moiety interacts with R209 and H169 via hydrophobic and hydrogen bonding interactions, and also in hydrophobic interactions with Y170 and W174. The reducing end of the H-type I glycan is further stabilized through a set of interactions between the Glc5 moiety with the residues H169, N171, S172, W174, and Y187. The carbon atom C6 of the SeFuc moiety is located within 3.9 Å of the side chain of R209 and forms a hydrophobic interaction with R209.

To examine whether binding of H-type I glycan causes structural changes in P[6] VP8*, we overlaid the unliganded and liganded P[6] VP8* structures (Fig. 3b). Although the overall structures superimpose well with a root mean square deviation (rmsd) of 0.71 Å for the matching Cα atoms, there are some noticeable changes. In the complex structures, the sidechains of Y170 and N171 change orientation to engage in a hydrophobic interaction with Gal4 and a hydrogen bond interaction with Glc5 of the glycan, respectively. In addition, the side chain of R209, that forms a hydrogen bond with E212 in the apo structure, binds to GlcNAc via hydrogen bond and hydrophobic interactions in the structure of P[6] VP8*/H-type I complex.

### Conserved residues interact with type I precursor motif

**Fig. 4:**
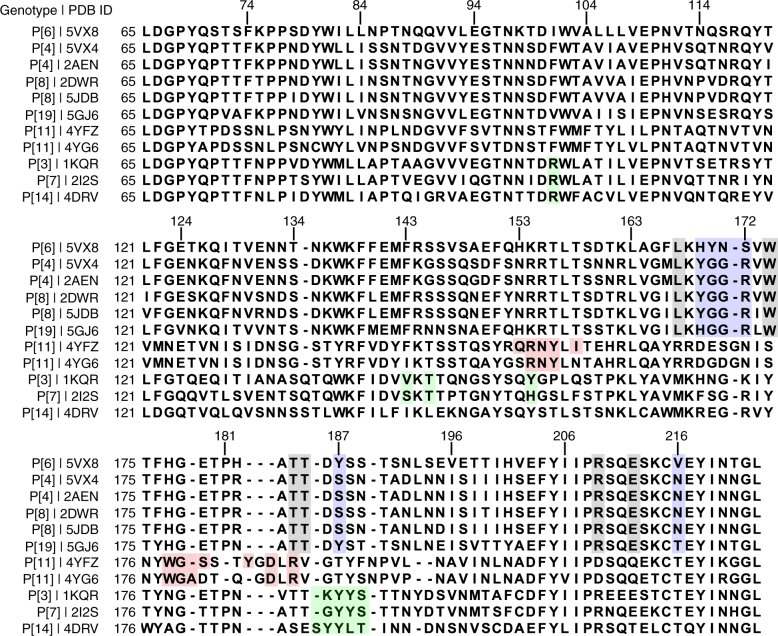
Structure-based sequence alignment of VP8*s in Chimera. The residues known to bind glycans are indicated with colored shade. The glycan binding amino acids that are conserved or non-conserved in the prevalent strains (P[6], P[4], P[8], and P[19]) are denoted with black and blue shades, respectively. The precursor glycan binding sites in P[11] RVs are labeled with light red shade. The A-type HBGA binding site in P[14] RV, and the sialic acid binding residues in P[3] and P[7] RVs are denoted with green shade. The PDB IDs for each structure are shown by the sequences

To examine whether the residues that interact with type I glycan in the P[6] VP8* are conserved and similarly disposed in the VP8*s of the other prevalent HRV genotypes P[4] and P[8], we superimposed the structure of P[6] VP8* in complex with H-type I with the previously published crystal structures of the native P[4] and P[8] VP8*s (Fig. 3c, d). The residues L167, W174, T184, T185, R209, and E212 that constitute the binding pocket for type I precursor (Galβ1,**3**GlcNAc) in P[6]VP8* are conserved in both P[4] and P[8] VP8*s (Figs. 3c, d and 4), suggesting that P[4] and P[8] VP8*s may also be able to engage the type I glycan via the precursor moieties. However, there are significant changes in the residues that recognize the Gal-Glc at the reducing end of the glycan. For example, H169 in P[6] VP8*, which forms a hydrogen bond with Gal4, is replaced by Y169 in P[4] and P[8] VP8*s. The residues Y170, N171, and Y187, with bulkier side chains in the P[6]VP8*, are changed to G170, G171, and S187 in P[4] and P[8] VP8*, leading to a loss of the hydrophobic interactions with the Galβ1,4Glc moieties. Further, S172 is replaced by R172 in VP8*s of P[4] and P[8] HRVs, which would provide steric hindrance to the type I glycan (Supplementary Fig. 4). These results indicate that P[4] and P[8] HRVs may bind to precursor moieties (Galβ1,**3**-GlcNAc) of the Type I glycan, but are likely to use a different mechanism to stabilize the Gal-Glc moieties at the reducing end of the glycan.

### P[4] and P[6] HRVs bind type I glycan in the same site

**Fig. 5:**
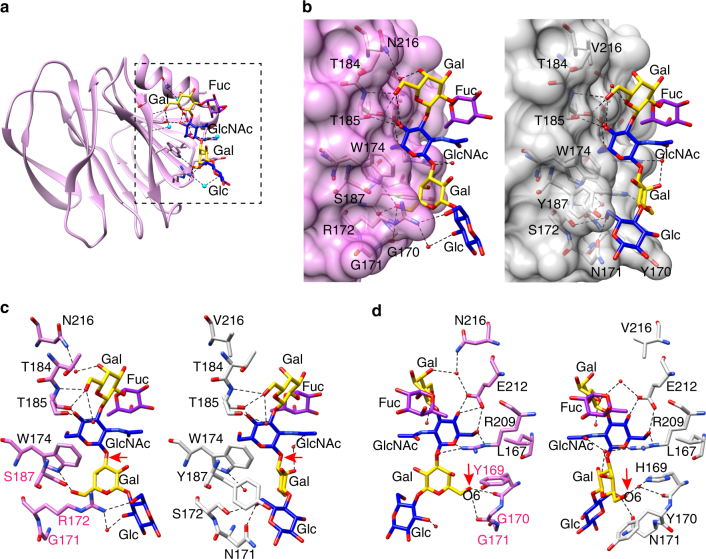
Structure of P[4] Indian VP8* in complex with H type I pentasaccharide. **a** Structure of P[4] VP8* is shown in pink ribbon with the bound glycan in stick model. The glycan residues are labeled. **b**–**d** Superimposition of the structures of P[4] VP8* (pink)/H-type I and P[6] VP8* (gray)/H-type I. **b** VP8* in surface representation shows the glycan binding pockets in P[6] and P[8]. **c**, **d** The glycan binding residues in P[4] and P[6] are shown in stick model in two views. The rotation of the glycosylic bond between GlcNAc and Gal is indicated with red arrows in **c**. The interactions between the side chain of H169 in P[6] VP8* and the O6 atom of Gal4, and that of the main chain carbonyl oxygen of Y169 in P[4] VP8* with the oxygen atom O6 of the Gal4 moiety are indicated by red arrows in **d**

To investigate how other prevalent HRV strains recognize type I glycans, we determined the crystal structure of VP8* of a G2P[4] HRV alone and in complex with H-type I pentasaccharide at 1.6 and 1.3 Å, respectively (Fig. 5a, Supplementary Fig. 2B and Table 1). The crystal structure of P[4] VP8* bound to H-type I glycan shows that the precursor (Galβ1,**3**-GlcNAc) interacts with VP8* at the same binding site as observed in the P[6] VP8* (Fig. 5b–d and Supplementary Fig. 3B). Most of the residues engaging the precursor moiety, such as T184, T185, E212 and R209, are not only conserved in the VP8*s of P[4] and P[6] but also in the other prevalent P[8] genotype. The only exception is Asn at position 216, which is conserved in P[4] and P[8] but is changed to Val in P[6]. While V216 in P[6] engages only in a hydrophobic interaction with Gal2, N216 in P[4] additionally participates in a water-mediated hydrogen bond interaction with Gal2 (Fig. 5c, d). The precursor and SeFuc moieties display similar configurations when bound on both P[4] and P[6] VP8*, indicating that the predominant HRVs use the same mechanism to recognize the precursor moieties in the type I glycans (Fig. 5b).

The presence of conserved residues in P[8] VP8* indicates that this genotype also contains the type I glycan binding site (Fig. 4). This is consistent with H-type I binding of P[8] VP8* observed using saliva- and oligosaccharide-based binding assays^37,41^. The crystal structure of VP8* from a P[8] HRV strain (Wa) has previously been determined, with two glycerol molecules bound on the protein^20^. Interestingly, one of the glycerol molecules bound on P[8] VP8* mimics the interactions observed with the GlcNAc moiety in the P[4] VP8*/H-type I complex (Supplementary Fig. 5A). The oxygen atoms O3 and O2 of glycerol, replace O4 and O6 in GlcNAc and form direct hydrogen bonds with side chains of T185 and E212 (Supplementary Fig. 5). The carbon atoms of glycerol are involved in hydrophobic interactions with L167, Y169, W174, and E212 in P[8] VP8*. The molecular interactions between glycerol and P[8] VP8*, resembling that of GlcNAc binding to P[4] and P[6] VP8*s, further suggests that the predominant HRVs all interact with the type I glycan in a similar manner.

### H-type I HBGA inhibits infectivity of prevalent HRVs

**Fig. 6:**
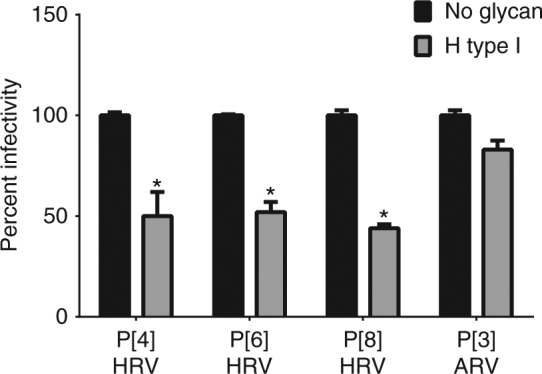
H-type I glycan inhibits infectivity of the prevalent HRVs. PAA-conjugated H-type I (1 mg/ml) significantly reduced the infectivity of P[4], P[6], and P[8] HRVs but not of a P[3] animal rotavirus (ARV, RRV strain). Each bar represents mean % infectivity, with no glycan treatment considered to be 100%. All assays were carried out a minimum of two times, with triplicates within each experiment. Error bars represent standard error of the mean. *P*-values < 0.05 were considered statistically significant [analysis of variance (ANOVA) with Sidak’s correction for multiple comparisons]

To investigate the biological relevance of H-type I binding to the prevalent HRVs, we performed fluorescent focus assays in the presence and absence of 1.0 mg/ml of PAA-conjugated multivalent H-type I glycan, with infections in the absence of glycan considered to be 100% infectivity. The PAA-conjugated H-type I glycan contains the common glycan residues, the type I precursor and the secretor Fuc, that are recognized by all prevalent HRVs. A significant reduction in infectivity was observed in the presence of H-type I-PAA for all three prevalent genotypes when compared to no glycan treatment (Fig. 6). In comparison, H-type I HBGA does not inhibit the infectivity of an animal rotavirus RRV strain (P[3] genotype) that recognizes sialic acid during cell entry^19^. In addition, we have performed infectivity assays using lactose (galactose and glucose with a β1,4 glycosidic linkage) at the same concentration as H-type I as a negative glycan control. Our results showed that lactose does not inhibit the infectivity of P[4], P[6], or P[8] rotaviruses (Supplementary Fig. 6). As lactose constitutes the two terminal residues at the reducing end of the H-type I pentasaccharide used in our structural studies, these results further indicate that the trisaccharide moiety at the non-reducing end of the H-type I is important for the interaction of H-type I with VP8*.

### Type 1 glycan bound to P[6] VP8* has altered orientation

**Fig. 7:**
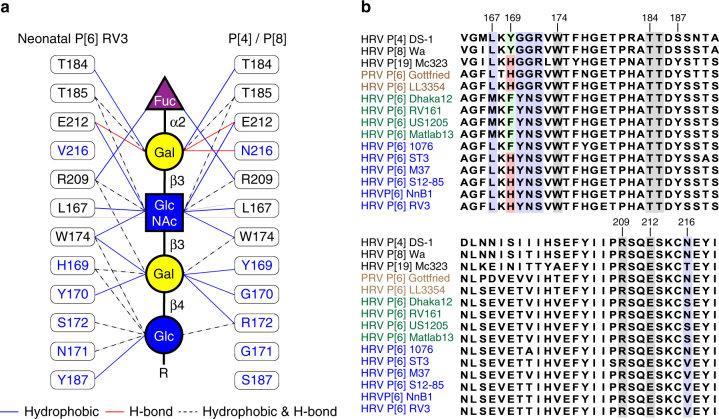
Summary of mutations at the glycan binding site in the prevalent strains. **a** The schematic diagram of glycan recognition in VP8*s of P[6], P[4], P[8], and P[19] genotypes that belong to the P[II] genogroup shows the common residues (black) interact with the Fuc-Gal-GlcNAc moieties, and the sequence changes (blue) lead to distinct recognition of the reducing end of the glycan in VP8*s. The molecular interactions between H type I glycan and P[4]/neonatal P[6] VP8*s are indicated by blue, red and black lines. The corresponding residues in P[6], P[8], and P[19] VP8*s are shown in the lateral columns. **b** Sequence alignment of VP8*s of the P[II] genogroup including those of porcine rotavirus (PRV) P[6], neonatal and non-neonatal P[6] HRV^16^. The conserved residues that recognize the type I precursor are indicated black shades, and other glycan binding residues are indicated by blue shade. The mutations at residue 169 are highlighted with green and red shades

Despite the similarity in the recognition of the precursor (Galβ1,3GlcNAc) moiety, VP8*s of the globally dominant HRVs display distinct differences in how they interact with the Galβ1,**4**Glc moiety at the reducing end of type I glycan (Fig. 5c, d). The orientation of the Gal residue in this moiety is entirely different between the P[6] and the P[4] VP8* structures. This change in orientation is caused by a single substitution involving H169 in the neonate-specific P[6] that is changed to Y169 in P[4] as well as P[8] VP8* (Figs. 5d and 7). In the neonate-specific P[6] VP8*, the side chain of H169 forms a hydrogen bond with the O6 atom of Gal3, whereas the main chain carbonyl oxygen of Y169 in P[4] VP8* forms a hydrogen bond with the oxygen atom O6 of the Gal moiety, resulting in a rotation of the glycosidic bond between the GlcNAc3 and Gal4 residues and leading to a distinct orientation of the reducing end of the glycan (Fig. 5d). Remarkably, sequence analyses reveal that the residue H169 is present in VP8* of most neonate-specific P[6] HRVs and it is substituted by F169 in the VP8* of P[6] HRVs that infect older children, with one exception, a neonatal strain 1076 containing F169. This change to Phe is similar to Y169 in P[4] and P[8] VP8* (Fig. 7).

As a result of the rotational changes in the glycosidic bond in the neonate-specific P[6] VP8*, the reducing end of the glycan is brought closer to VP8* establishing a number of hydrophobic interactions involving Y170, N171, and Y187 and hydrogen bond interactions involving N171 and S172 (Fig. 5c, d). In contrast, in the P[4]VP8* and possibly P[6] VP8* from older children, the reducing end of the glycan is essentially solvent exposed with minimal interactions with VP8*. The only interaction that the reducing end makes is a hydrophobic interaction with G170. In addition, the longer side chain of R172 in P[4]VP8*, which is a Ser residue in the P[6] VP8*, also keeps the reducing end away from the VP8* (Fig. 5c, d). These genotypic variations in the neonate-specific P[6]VP8* constrain the orientation of the reducing end of the type I glycan restricting its ability to bind branched glycans. This may be an important factor in conferring the age-restricted tropism of certain P[6] strains because precursor glycans and unbranched structures are more abundant in the neonatal gut and branching at the reducing end is developmentally regulated^35,42^

### Involvement of secretor fucose in glycan binding

*FUT2* secretor status is associated with RV infections as shown by recent epidemiology studies, suggesting that the SeFuc may be recognized by RV during virus attachment^30–34^. Our crystal structures of P[6] and P[4] VP8*s in complex with H-type I show that the SeFuc interacts with the residue R209 in both VP8*s (Figs. 3b and 5d and Supplementary Fig. 3). Compared to the number of interactions that the precursor moieties make with the VP8*, the involvement of the SeFuc is rather minimal. To examine if the SeFuc interacts with VP8* in solution, we determined the binding constant (*K*_d_) using NMR spectroscopy by monitoring the chemical shift changes as a function of increasing ligand concentration (Supplementary Fig. 7). Eleven representative NMR peaks displayed significant shift changes (^1^H dimension) in the 2D ^1^H-^15^N HSQC spectra and were globally fitted using Origin2016 (OriginLab, Northampton, MA) to get a *K*_d_ of 7.1 ± 0.2 mM (Supplementary Fig. 8; error estimated as the nonlinear least-squares fitting parameter standard error using the Levenberg–Marquardt algorithm). This estimated K_d_ is similar to that obtained for LNFP1 binding with P[19] VP8* using STD NMR^27^. In addition, we carried out NMR STD experiments to confirm that the SeFuc of H-type I interacts with VP8* as observed in the crystal structure. The STD spectra show that the methyl resonances of the fucose moiety in the glycan are present only in the presence of the protein, indicating that the SeFuc does interact directly with the protein in solution (Supplementary Fig. 9).

### Structures support binding of ABH but not Lewis HBGA

**Fig. 8:**
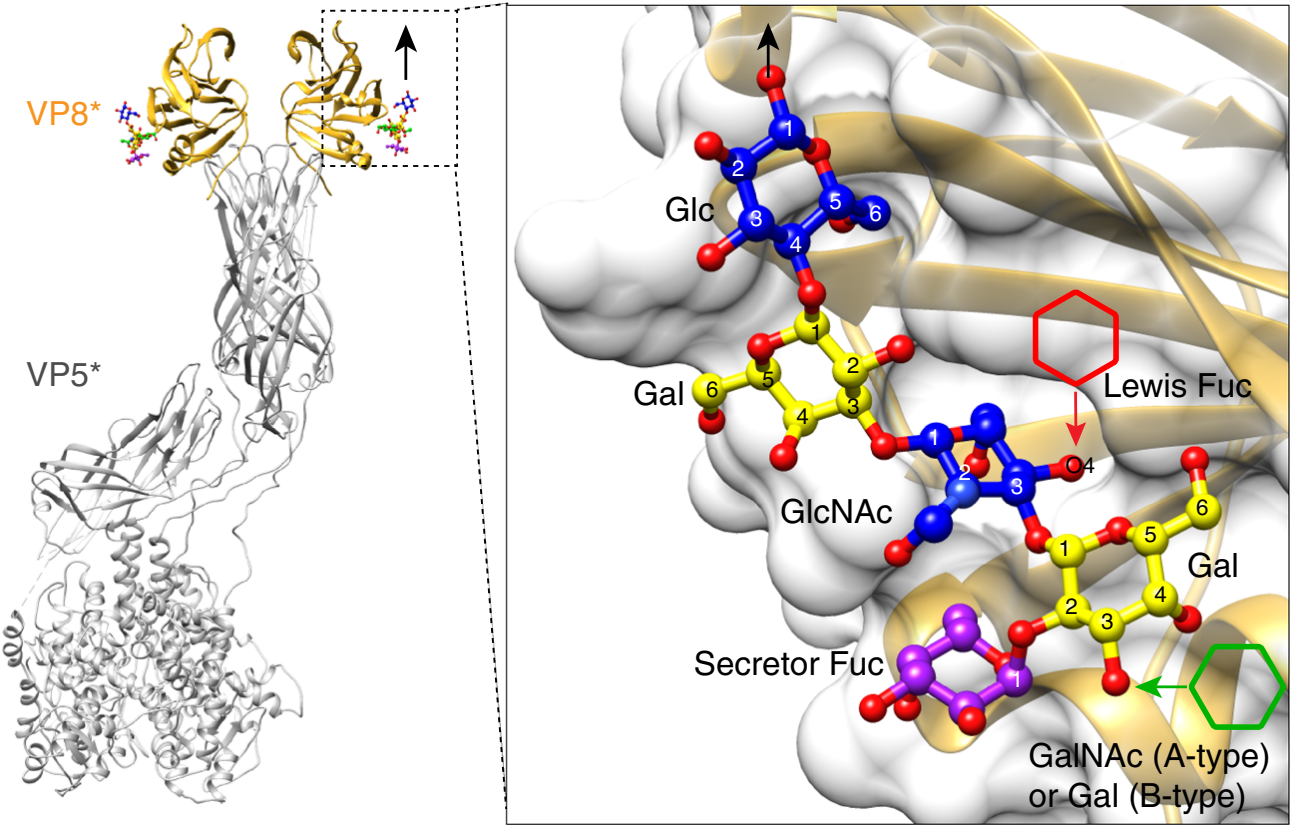
Structural basis for resistance to rotavirus infection in non-secretors. P[6] VP8* in complex with H type I pentasaccharide is superimposed onto the structure of VP4 spike (PDB ID: 4V7Q) of P[3] animal strain RRV. The black arrow indicated how the glycan is extended from the C1 atom of Glc residue, and how the virus spike access host cell membrane. A close-up view of the P[6] VP8* with H-type I is shown in the inlet. The addition of a Lewis fucose via an α1,4 linkage to the GlcNAc moiety is indicated by a red hexagon, suggesting that Lewis fucose would clash with VP8*. The addition of GalNAc or Gal at the non-reducing end of HBGA is noted by a green hexagon, which is projecting away from VP8* without making steric hindrance

The co-crystal structures of P[4] and P[6] VP8*s with H-type I HBGA provide a structural rationale for the recognition of A- or B-type HBGA in the newly identified glycan binding pocket in the globally dominant HRV strains. When the GalNAc residue in A-type, or the Gal in B-type HBGA, is added to the O3 atom of the Gal moiety of the H-type I at the non-reducing end, it projects into the solvent without making any steric hindrance, indicating that VP8*s of the prevalent HRV should be able to bind both A- and B-type HBGAs (Fig. 8). Since a recent epidemiology study also suggests the importance of Lewis HBGA status in infections with specific RV genotypes such as P[6]^33^, we modeled structural interactions of the globally dominant HRV VP8* with Lewis HBGA. The addition of LeFuc via α1,4-linkage to the O4 atom of the GlcNAc residue in the H-type I causes severe steric clashes (Fig. 8), suggesting that it is unlikely that the VP8* of the prevalent HRVs recognize Lewis HBGAs. This is consistent with recent studies on P[19] VP8*, which showed no binding to Lewis antigens^27^. Similarly, in type II precursor glycans, Gal residue linked to the O4 atom of the GlcNAc via a β1,**4** (type II) linkage would clash with P[4] and P[6] VP8*s, indicating that the prevalent strains prefer to bind type I glycans. The glycan binding site as revealed by our structural studies on P[4] or P[6] VP8* is also accessible in the context of the virion-bound VP4 spike structure, as demonstrated by the superimposition of VP8* structures with the bound H-type I pentasaccharide onto the atomic structure of the virion-bound VP4 spike (Fig. 8). This superposition shows that the glycan binding O1 atom of the terminal Glc residue at the reducing end points outwards from the spike, indicating that longer glycan chains bearing the type I backbone on the cell surface can access the binding site on VP8*.

## Discussion

Recent structural, biochemical, and epidemiological studies underscore the importance of HBGA glycans in the cell attachment and susceptibility to HRVs, similar to other gastric pathogens such as human noroviruses and *H. pylori*^18,43^. While structural evidence for HBGA interactions was previously characterized for unusual HRVs such as P[14] and P[11] strains, the structural basis for how the globally dominant HRV strains, which include P[4], P[6], and P[8] genotypes, interact with the HBGA has been unclear. The structure of the P[6] VP8* or the structures of the VP8*s of any of these genotypes in complex with a glycan have not been previously reported. Our structural studies have provided new insights into glycan recognition by these prevalent HRV genotypes. From these studies, we have shown that the VP8* of these HRV genotypes bind to type I glycans using a distinct site that can support the binding of precursor and ABH HBGAs but sterically prohibit the binding of Lewis HBGAs. The residues that constitute the binding site are highly conserved in these HRV VP8*s except for one critical change in the neonate-specific P[6]VP8* that profoundly alters the orientation of bound glycan that would be restrictive for branching at its reducing end. These observations are discussed below in relation to the glycan binding as observed previously in the VP8* of other RV strains, and also in the context of the available epidemiological studies.

In the VP8* of the P[14] HRV, which specifically recognizes A-type HBGA, the glycan binding site is located at one corner of the cleft between the two β-sheets overlapping with the sialic acid binding site in the VP8* of the P[3] ARVs^23^. In the VP8* of the P[11] RVs, the glycan binding site is expansive, extending across the cleft region of the galectin fold^24^. In the VP8* of the P[4] and P[6] genotypes, the glycan binding site is in an entirely different location between the C-terminal helix and one of the two β-sheets of the VP8* (Fig. 3 and Fig. 5). This is consistent with the recent NMR and crystallography studies on P[19] VP8* showing binding to H-type I pentasaccharide (LNFP1) in a similar location as that observed in our studies^27,36^. The P[19] genotype belongs to the same genogroup as P[4], P[6], and P[8]. Similarities in the glycan specificity and binding between the prevalent HRVs and the P[19] RV, which primarily infects pigs, perhaps reflects a zoonotically related evolution of these strains. More recently, the apo crystal structure of porcine P[6] strain (z84) has been reported, showing a very similar strutcure as P[19] VP8*^44^. Interestingly, H-type I is the dominant glycan in the porcine gut^45,46^. Further, superposition of the P[4] and P[8] VP8* structures strongly suggest that the P[8] VP8* can bind to HBGAs in the same manner as observed in our P[4] and P[6] VP8* crystal structures. Considering that the P[8] Wa strain has been previously shown to recognize a sialoglycan GM1a^21^, the possibility that the globally dominant HRVs recognize other cellular glycans, in addition to HBGA-related glycans, during the entry process should not be discounted.

Among the prevalent HRV genotypes, the P[6] genotype was historically thought to be primarily associated with neonatal infections^47^. Subsequent epidemiological studies have revealed that although P[6] infections are less common globally when compared to P[8] and P[4] genotypes, infections with P[6] strains in infections in children under the age of 5 occur in certain geographic regions^16,48^. The P[6] VP8* in our X-ray crystallography and NMR studies is from the neonate-specific HRV vaccine strain RV3. Our structural studies provide a possible structural rationale for the neonate specificity observed in some P[6] HRV strains. In the neonate gut, modification to the precursor moieties or the branching at the reducing end is developmentally regulated^35,42^. While ABH and Lewis HBGAs are also present, the most abundant type in the neonate gut is the unbranched type I precursor glycans^35,49^. Remarkably in the neonatal P[6] VP8*, a single residue H169, which is well conserved in most neonate-specific [P6] genotypes, alters the conformation of the glycan to sterically restrict branching at the reducing end. In the other HRV genotypes that are not restricted to neonates, this residue is mutated to either Tyr or Phe, allowing the reducing end of the glycan to be solvent exposed and suggesting that these genotypes can bind more complex branched glycans. Further, despite significant differences in the binding sites between neonate-specific P[11] and the P[6] VP8*, the conformation of the bound glycan is very similar. In the neonate-specific P[11] VP8*, the conformation of the bound glycan does not favor binding of glycans that are branched at the reducing end.

Recent epidemiological studies have overwhelmingly shown that Se+ individuals are significantly more susceptible to infection by the prevalent HRV P[8] genotype^30–34^. Consistent with these findings, our structural studies demonstrate that VP8* of the globally dominant P[4], P[6], and P[8] genotypes can bind to all ABH HBGAs. An expected feature of such a correlation is the involvement of the SeFuc in the interactions with the VP8*. Involvement of the SeFuc in P[II] genogroup VP8* has been shown through crystallography and NMR in this study, and also by previous NMR solution studies on P[4], P[6], and P[19] VP8*s^27,28^. These studies show that the C6 atom of SeFuc interacts with R209, which is conserved in all the strains in the P[II] genogroup, consistent with previous NMR studies with P[4] DS-1 and P[6] RV3^28^. However, nominal interactions involving the SeFuc suggest that this moiety does not contribute significantly to the binding affinity and that VP8* should be able to bind to the type I precursor with a reasonably strong affinity. The implication then would be that non-secretor individuals can be susceptible to infections with prevalent HRV strains. Since most epidemiological evidence thus far has been from studies on secretor status and severe RV diarrhea requiring hospitalization, it is possible that non-secretors are susceptible to infection with prevalent HRVs. This appears to be true both in vitro, where recently it was shown that human intestinal enteriod cultures made from Se− individual can be infected by P[8] genotype^50^ and in the epidemiological study which shows secretor status was not significantly associated with susceptibility to P[4] and P[6] RV infections^51^.

Our finding that the presence of LeFuc is not favorable for binding is seemingly in contradiction to the epidemiological differences in genotype distribution between Le+ and Le− individuals^33^. It is to be noted that typically not all type I precursors (in non-secretors) or ABH HBGAs (in Se+ individuals) are entirely converted to Lewis HBGAs in intestinal epithelial cells^49,52^. The likely presence of precursor or the ABH HBGAs together with Lewis HBGAs may be the reason for the susceptibility in such individuals.

An interesting observation from the epidemiological studies is that in comparison to P[4] and P[8] HRVs, P[6] HRVs have a restricted geographic prevalence and are more common in sub-Saharan Africa^9^. In these countries, the Le-negative phenotype is more common among the population than in other geographic locations^9,33^. It has been suggested that Le− children are susceptible to infections with P[6] viruses but not P[8], and this may contribute to epidemiological differences in this population as well as the poor efficacy of the current RV vaccines, which are attenuated P[8] strains^33^. Based on our structural studies, all the prevalent strains can bind either the precursor (Se−Le−) or ABH HBGAs (Se+Le−), contrasting with the data from field studies. Although the glycan specificity is an important factor for infectivity, the relative distribution of RV genotypes and vaccine efficacy in different populations may be impacted by several other factors, including subtle variations in the glycan binding affinities between the genotypes, the presence or absence of co-receptors, differential immune responses, and other host factors^53^. Further studies are necessary to examine how these factors intersect with glycan specificity and impact the prevalence of a particular genotype or the efficacy of a vaccine.

In summary, our studies underscore the emerging theme that the glycan recognition in RVs is far more complex than previously envisioned and that they exhibit enormous genotype-dependent glycan specificity by encoding the binding sites in distinct regions of VP8* within the same framework of a conserved galectin-like fold. Such variation in glycan recognition mechanisms is entirely consistent with the species tropism, zoonosis, adaptation and epidemiological prevalence observed among RVs. Our studies here provide new structural data that were not previously available as to how the globally dominant HRV genotypes recognize type I glycans, which are the most abundant glycans in the intestinal epithelial cells. A surprising finding from our studies is that all the prevalent genotypes share a common glycan binding site that can be exquisitely fine-tuned by subtle genotypic variations to accommodate the age-dependent propensity of the unbranched and more complex glycans. In all these genotypes, the region within this binding site that interacts with the precursor moieties is highly conserved and can be an attractive target for drug design. Thus, this can provide a rational basis for designing small molecules that block glycan binding as antiviral drugs, and perhaps even for the formulation of more effective vaccines.

## Methods

### Protein expression and purification

Recombinant N-terminal GST-tagged VP8*s of G3P[6] HRV neonate-specific RV3 (Accession ADD31861) and an Indian G2P[4] HRV (PDB ID: 5VX4) were expressed in *E. coli* BL21(DE3) cells (Novagen) and purified with Glutathione Sepharose 4 Fast Flow (GE healthcare) affinity column. The GST tag was removed by the treatment with protease thrombin overnight at 4 °C, and rebinding the protein mixture to the Glutathione Sepharose column. The VP8* was further purified by size exclusion column Superdex75 (GE healthcare) with 10 mM Tris, pH 7.4, 100 mM NaCl, 1 mM dithiothreitol (DTT) at 4 °C. The concentration of the purified protein was determined by measuring absorbance at 280 nm and using an absorption coefficient of 32,430 per M per cm for both VP8*s calculated using ProtPraram on the ExPASy server^54^.

### Crystallization

Crystallization screenings for the P[4] and P[6] VP8*s at the concentration of 16 mg/ml were carried out by hanging-drop vapor diffusion using the Mosquito crystallization robot (TTP LabTech) and imaged using Rock Imager (Formulatrix) at 20 °C. Both VP8*s were crystallized alone or co-crystallized with 40 mM H-type I pentasaccharide (LNFP1) purchased from Dextra Labs. The unliganded P[6] RV3 VP8* was crystallized under the condition with 1.7 M ammonium sulfate, 0.1 M Tris, pH 7.0. The RV3 VP8*/LNFP1 co-crystals were obtained from the condition with 2.0 M ammonium sulfate, 0.1 M Tris, pH 8.5. The unliganded Indian G2P[4] VP8* was crystallized under the condition with 0.1 M Bis-Tris propane, pH 9.0, 30% w/v PEG6000, and the P[4] VP8*/LNFP1 complex was crystallized with 0.2 M sodium acetate, 0.1 M Tris, pH 8.5, 30% w/v PEG4000. Crystals were flash-frozen directly in liquid nitrogen.

### Structure determination and refinement

Diffraction data for the unliganded and liganded P[4] and P[6] VP8*s were collected on 5.0.1 Beamline at Advanced Light Source, Lawrence Berkeley National Laboratory. Diffraction data were processed with IMOSFLM as implemented in the CCP4 suite^55^. The P[4] DS-1 VP8* native structure (PDB ID: 2AEN) was used as a search model for molecular replacement (MR) using PHASER. Automated model building and solvent addition were carried out using ARP/wARP^56^. The atomic model including the side chain atoms obtained from ARP/wARP was then subjected to iterative cycles of refinement using PHENIX and further model building using COOT based on the difference maps^57,58^. The LNFP1 was generated using the SWEET2 package of the Glycosciences.de server (http://www.glycosciences.de) and modeled into the electron density using COOT. The density and stereochemistry of the glycans, and the conformational changes in the VP8* were validated by computing simulated annealing omit maps using PHENIX and the CARP package^59^. Data collection and refinement statistics following final refinement cycle are given in Table 1. Ligand interactions were analyzed using LigPlot^+^^60^. The structural alignments and calculations of rmsd were carried out using the Chimera^61^. Figures were prepared by using Chimera.

### NMR spectroscopy

^1^H-^15^N-labeled P[6] RV3 VP8* was purified as described previously. P[6] VP8* and LNFP1 ligand were dissolved in 10 mM sodium phosphate, 50 mM NaCl, 10% D_2_O buffer, pH 7. NMR experiments were performed on Avance III HD 800 MHz Ascend™ Bruker instrument equipped with quadruple resonance inverse detection QCI CryoProbe^TM^ (NMR and Drug Discovery Core, Baylor College of Medicine).

### *K*_d_ determination

2D ^1^H-^15^N HSQC (heteronuclear single quantum coherence) NMR experiments were collected on P[6] VP8* (70 μM) using various ligand (LNFP1) concentrations (0.5, 2.0, 3.5, 5, 8, 20 mM). NMR spectra were analyzed using NMRPipe^62^ and NMRFAM-Sparky^63^ software. Chemical shift changes in the ^1^H dimension for 11 peaks (Supplementary Figs. 7 and 8) were used to calculate the binding constant (*K*_d_). The data were globally fit to the general binding model “*ML* ↔ *M* + *L*” using the equation

$$Y = \left( {\left[ {ML} \right]/\left[ {M_{\mathrm{T}}} \right]} \right)\left( {Y_{{\mathrm{ML}}}-Y_{\mathrm{M}}} \right) + Y_{\mathrm{M}}$$

*M*_*T*_ is the total *M* concentration independent of ligation state, *Y* is Δδ^1^H, *Y*_*M*_ and *Y*_*ML*_ are the binding transition baselines, and [*ML*] = (−*b*−(*b*^2^−4*ac*)^0.5^)/2*a*, with *a* = 1, *b* = −*K*_d_−[*M*_T_]−[*L*_T_], and *c* = [*M*_T_][*L*_T_], as described previously^64^.

### STD NMR experiments

STD experiments were carried out with 70 μM P[6] VP8* and 8 mM LNFP1 (1:116 protein:ligand ratio), and 86.7 μM Vp8 and 5.2 mM LNFP1 (1:60) acquired at 10 °C and 25 °C using pulse sequence *stddiffgp19*. The protein resonances were saturated at 0.2 ppm (also at −1 and 0 ppm, with similar results), with 2 s saturation time. The off-resonance saturation was applied at 30 ppm and a total of 1024 scans were collected with a 5 s recycle delay. A spin-lock filter with 100 ms duration was applied to suppress the broad protein resonance signals, and watergate 3-9-19 to suppress residual water signal.

### Infectivity assays

Infectivity assays to determine the effect of glycans were performed using fluorescent focus assays on African green monkey kidney epithelial cells (MA104 cells) seeded onto 96-well plates and laboratory-adapted P[3], P[4], P[6], and P[8] rotavirus strains^65^. The MA104 cells and P[3], P[4], and P[8] rotaviruses were serially passaged in the Estes laboratory at Baylor College of Medicine^66^. The P[6] rotavirus strain was kindly provided by Monical McNeal (Cincinnati Childrens Hospital, OH). Briefly, dilutions of virus yielding ~100–200 focus forming units per well were preincubated for 30 min with media containing PAA-conjugated H-type I (Glycotech) or media without glycan. The H type I was tested at a concentration of 1 mg/ml based on findings from a study on P[19] RVs where this concentration showed the highest inhibitory effect^36^. After cooling the cells and virus to 4 °C, the inoculum was added and allowed to bind to cells on ice for 1 h. The inoculum was then removed and cells were washed once with ice-cold DMEM to remove unbound virus. Media with or without H-type I-PAA was added to the cells and infections were allowed to continue at 37 °C for 15 h. The cells were then fixed with ice cold methanol. Infected cells were detected with an anti-rotavirus polyclonal rabbit primary antibody (raised in-house, 1:300 dilution)^66^ followed by a fluorescently conjugated anti-rabbit secondary antibody (ThermoFisher Scientific, 1:1000 dilution). Infections in the absence of glycan served as controls and were considered to be 100% infectivity. Statistical comparisons were carried out using Student’s t-test, with *p*-values < 0.05 considered as statistically significant. The same approach was followed for the evaluation of P[4], P[6] and P[8] HRV infectivity using lactose as a glycan control, at the same concentration as H-type I HBGA.

### Data availability

The coordinates and structure factors of the native P[6] VP8* and its complex with LNFP1 have been deposited in the Protein Data Bank (www.pdb.org) with the accession codes 5VX8 and 5VX9, respectively. And the accession codes for the native P[4] VP8* and its complex with LNFP1 are 5VX4 and 5VX5, respectively. The authors declare that all other data supporting the findings of this study are available within the article and its Supplementary Information files, or are available from the authors upon request.

## Electronic supplementary material

Supplementary Information
