# Supplementary material for: Glycan recognition in globally dominant human rotaviruses
Source: Nat Commun. 2018 Jul 6;9:2631. doi: 10.1038/s41467-018-05098-4 (PMC6035239; doi:10.1038/s41467-018-05098-4)
Supplement: Supplementary file 1 — Supplementary Information [file 41467_2018_5098_MOESM1_ESM.pdf]

SUPPLEMENTARY INFORMATION

Glycan Recognition in Globally Dominant Human Rotaviruses

Hu et al.

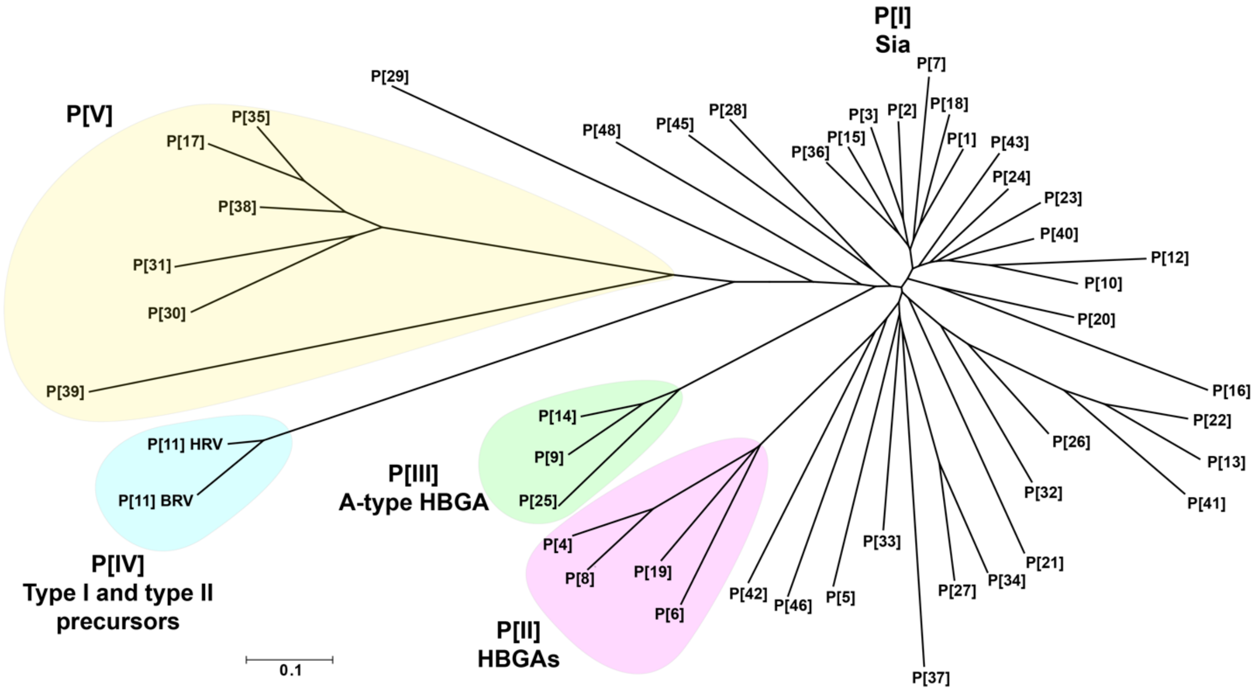

Supplementary Figure 1. Phylogenetic analysis of VP8*s from 46 P genotypes. The maximum-likelihood analysis was carried out using MEGA6[1].

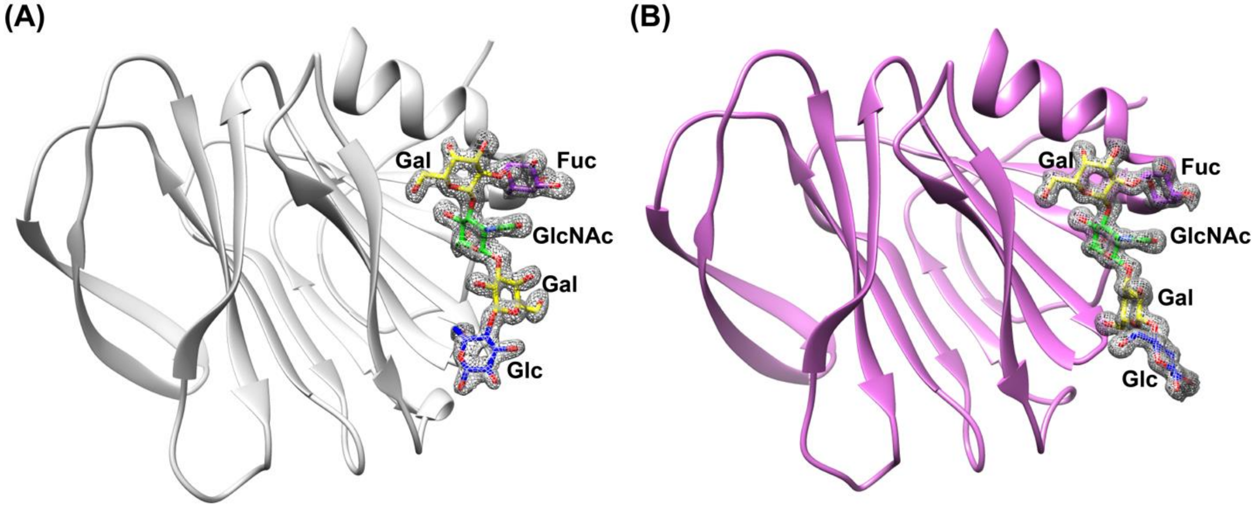

Supplementary Figure 2. Electron density map of LNFP1 pentasaccharide bound on P[6] RV3 VP8* (A) or P[4] Indian VP8* (B). The unbiased simulated omit electron density maps (Fo-Fc) for the bound glycan receptors contoured at 3.0 sigma. The Fo-Fc maps are generated by FFT program in CCP4 software[2]. H-type I is shown in stick model with the β-D-Galactose (Gal), the N-acetyl-D-glucosamine (GlcNAc), the β-D-Glucose (Glc) and the α-fucose (Fuc) colored in yellow, green, blue and purple, respectively.

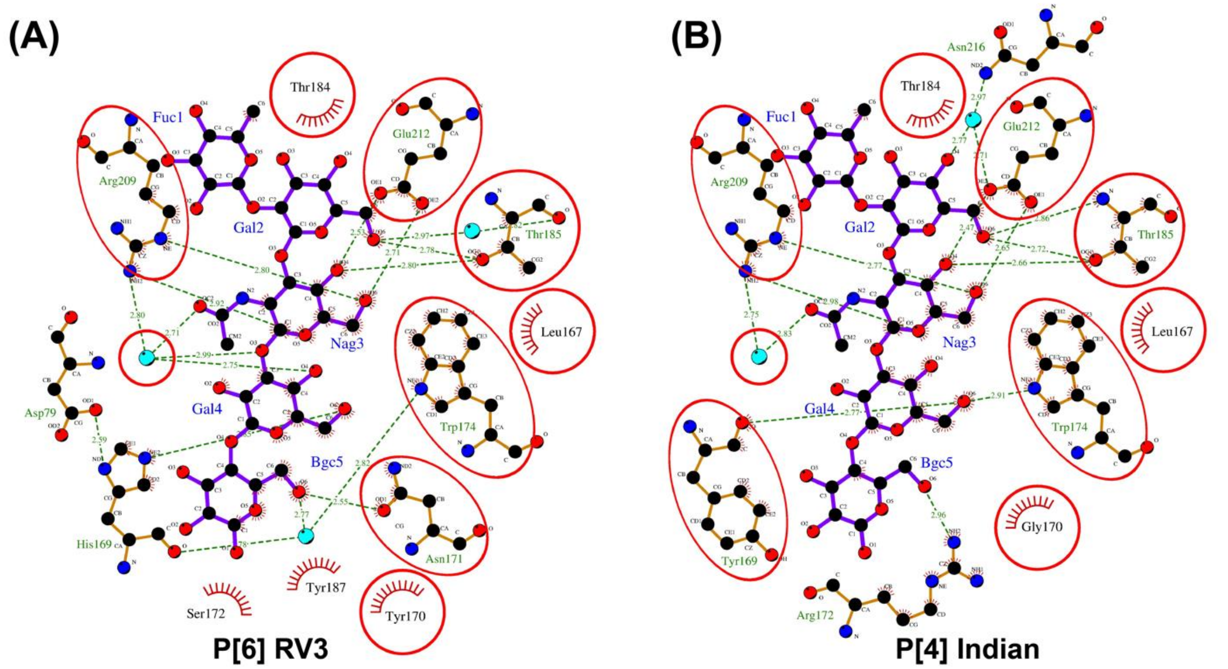

Supplementary Figure 3. Interactions between H type I pentasaccharide and P[6] RV3 VP8* (A) or P[4] Indian VP8* (B) as determined using LIGPLOT+[3]. The structures are superimposed and shown side by side.

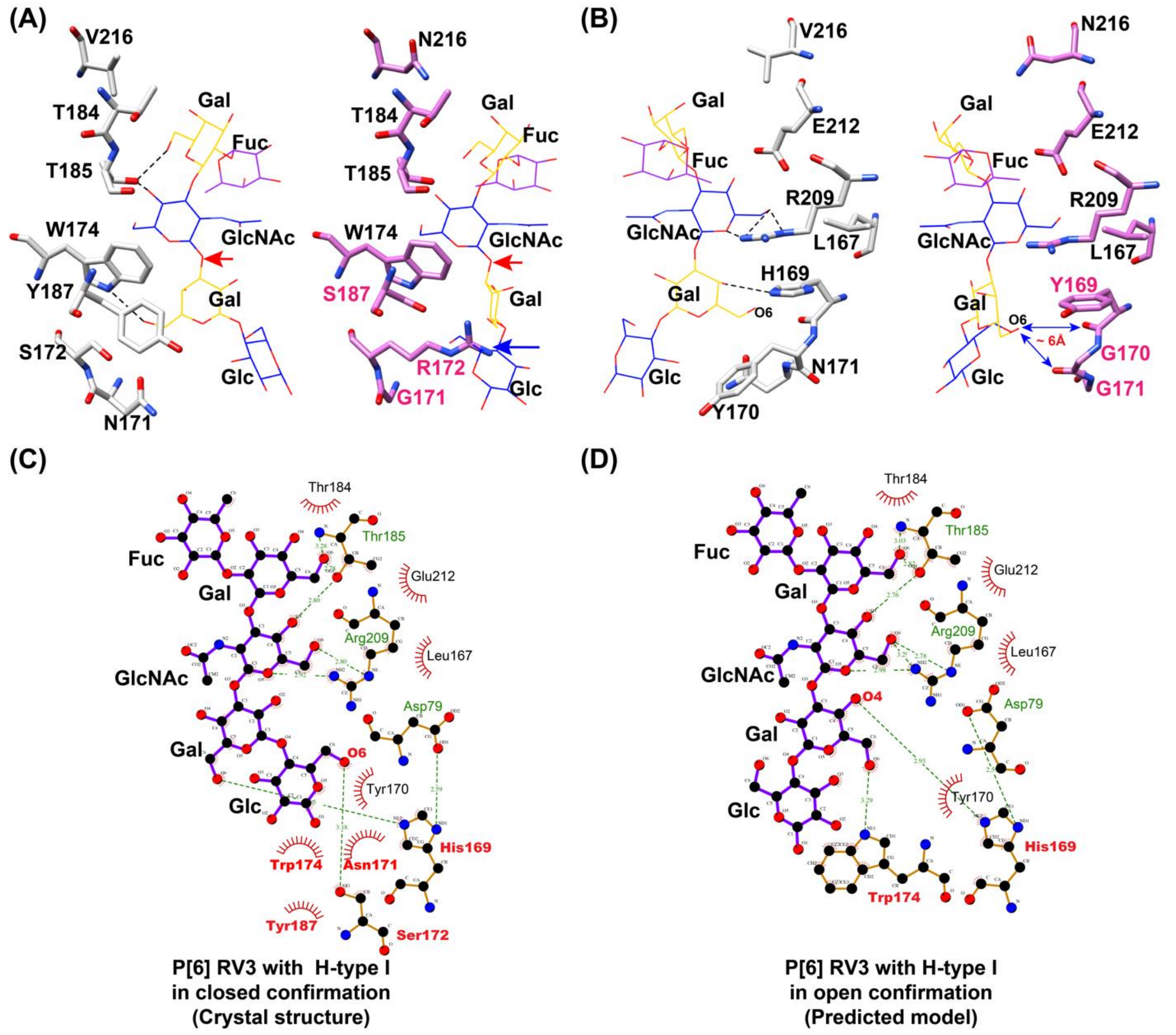

Supplementary Figure 4. Structural analysis of P[6]/H-type I and P[4]/H-type I complexes. (A)-(B) Glycan binding residues in P[6] are shown grey sticks and the type I glycan in the orientation that is observed in P[4] VP8*("open" confirmation) is shown as wire; Glycan binding residues in P[4] are shown pink sticks and the type I glycan in the orientation that is observed in P[6] VP8* ("closed" confirmation) is shown as wire. The blue arrow indicates that the side chain of R172 in P[4] VP8* will clash with type I glycan in "closed" confirmation. (C) Ligplot of P[6] VP8* with H-type I in the closed confirmation as observed in the crystal structure and (D) ligplot

of P[6] with H-type I in a modeled open confirmation, showing that the reducing end of H-type I in the open confirmation loses several interaction with P[6].

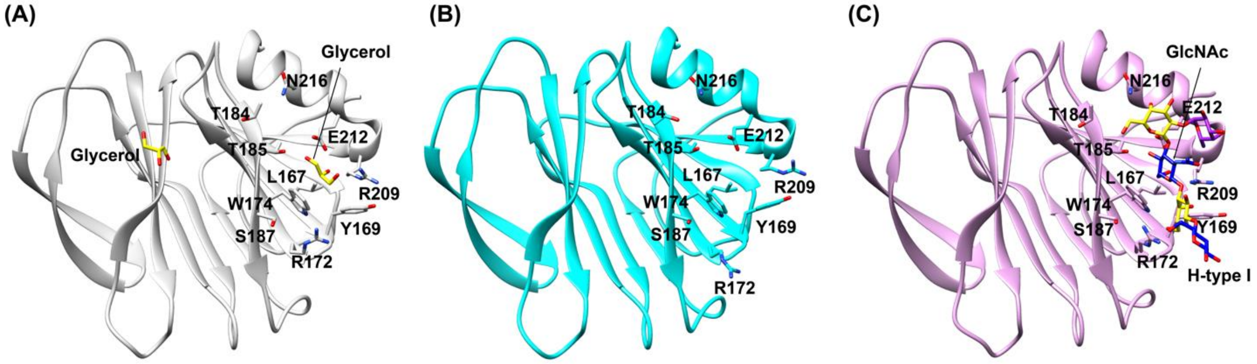

Supplementary Figure 5. Structural comparison of P[8] Wa (grey), P[8] Indian (cyan) and P[4]/H-type I complex (pink) reveals the conserved potential glycan binding site on P[8] VP8*s. (**A**), Structural alignment of VP8* of P[8] Wa (grey) in complex with two glycerol molecules (yellow stick) with P[8] Indian (cyan) or P[4] Indian VP8* (pink) in complex with H-type I. For clarity, the structure of P[8] Indian (cyan) and P[4] Indian VP8 (pink)/H-type I is shown side-by-side in (**B**) and (**C**), respectively. H-type I pentasaccharide is shown in stick model.

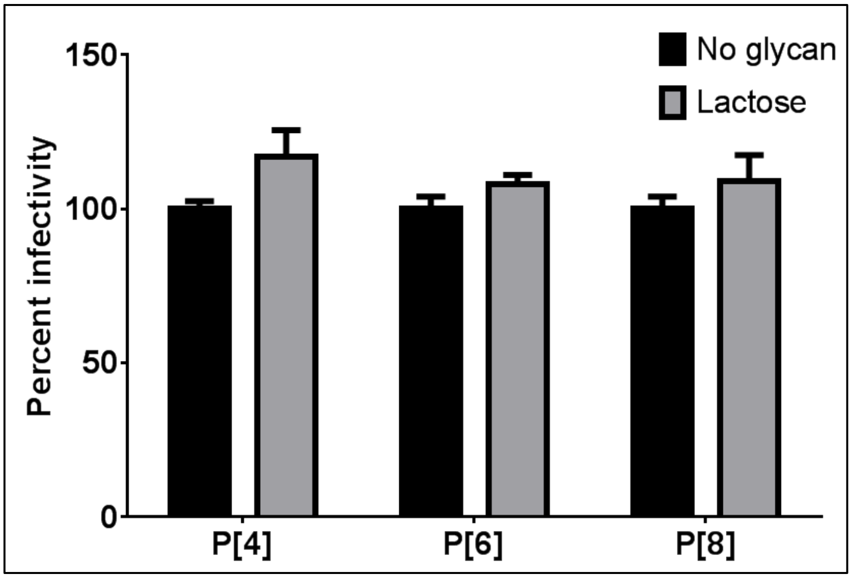

Supplementary Figure 6. Lactose does not inhibit the infectivity of prevalent HRVs. The infectivity of P[4], P[6] and P[8] HRVs was not inhibited by lactose (1 mg/ml) used as a control glycan. Each bar represents mean % infectivity, with no glycan treatment considered to be 100%. All assays were carried out a minimum of two times, with triplicates within each experiment. Error bars represent standard error of the mean. P-values<0.05 were considered statistically significant (Analysis of variance with Sidak's correction for multiple comparisons).

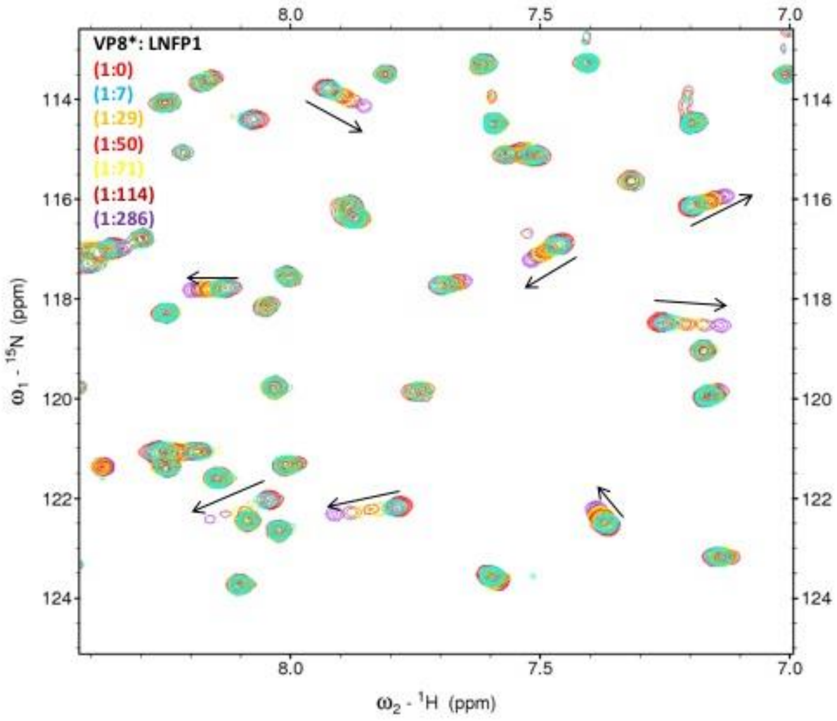

Supplementary Figure 7. NMR-detected specific interactions P[6] RV3 VP8* between H type I pentasaccharide. A spectral region of 2D ^{1}H-^{15}N HSQC spectra of ^{15}N labeled P[6] RV3 VP8* alone (red) and in the presence of various ligand concentrations of glycan LNFP1 (0, 0.5, 2, 3.5, 5, 8, 20 mM; mole ratio of VP8*:LNFP1 is 1:0 - 1:286; red to purple) showing several peaks with increasing chemical shift movements (arrow) with increasing ligand concentrations.

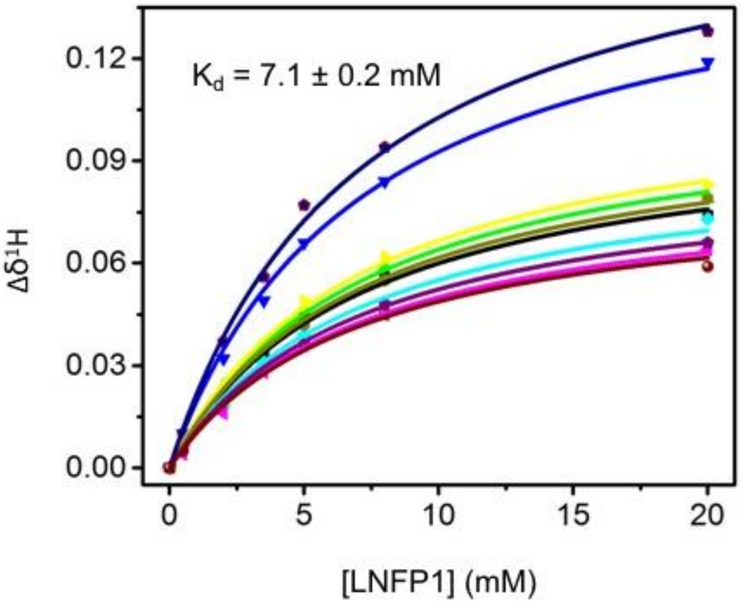

Supplementary Figure 8. **NMR-derived binding affinity between P[6] RV3 VP8* and H type I pentasaccharide.** Binding curves of NMR titrations of LNFP1 to P[6] RV3 VP8* for 11 representative NMR peaks (using changes in proton chemical shifts). The data were globally fitted to get a binding affinity K_d of 7.1 ± 0.2 mM.

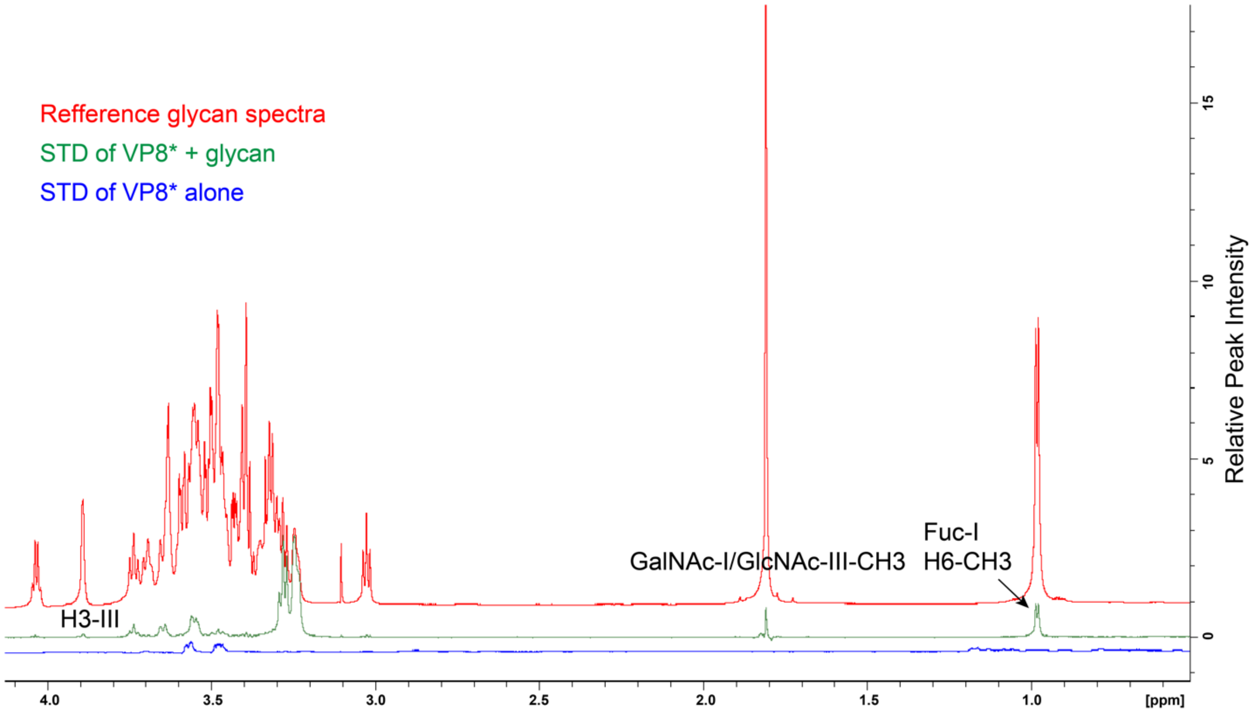

Supplementary Figure 9. Direct interaction between P[6] RV3 VP8* and the SeFuc moiety of H type I pentasaccharide. 1D ^{1}H NMR spectra of LNFP1 in complex with P[6] RV3 VP8* (60:1 molar ratio), showing the binding of fucose moiety to VP8* in the saturation transfer difference (STD) spectra (middle, green), with the reference (off-resonance) spectra on the top (red). The spectra of VP8* protein alone is shown in blue.

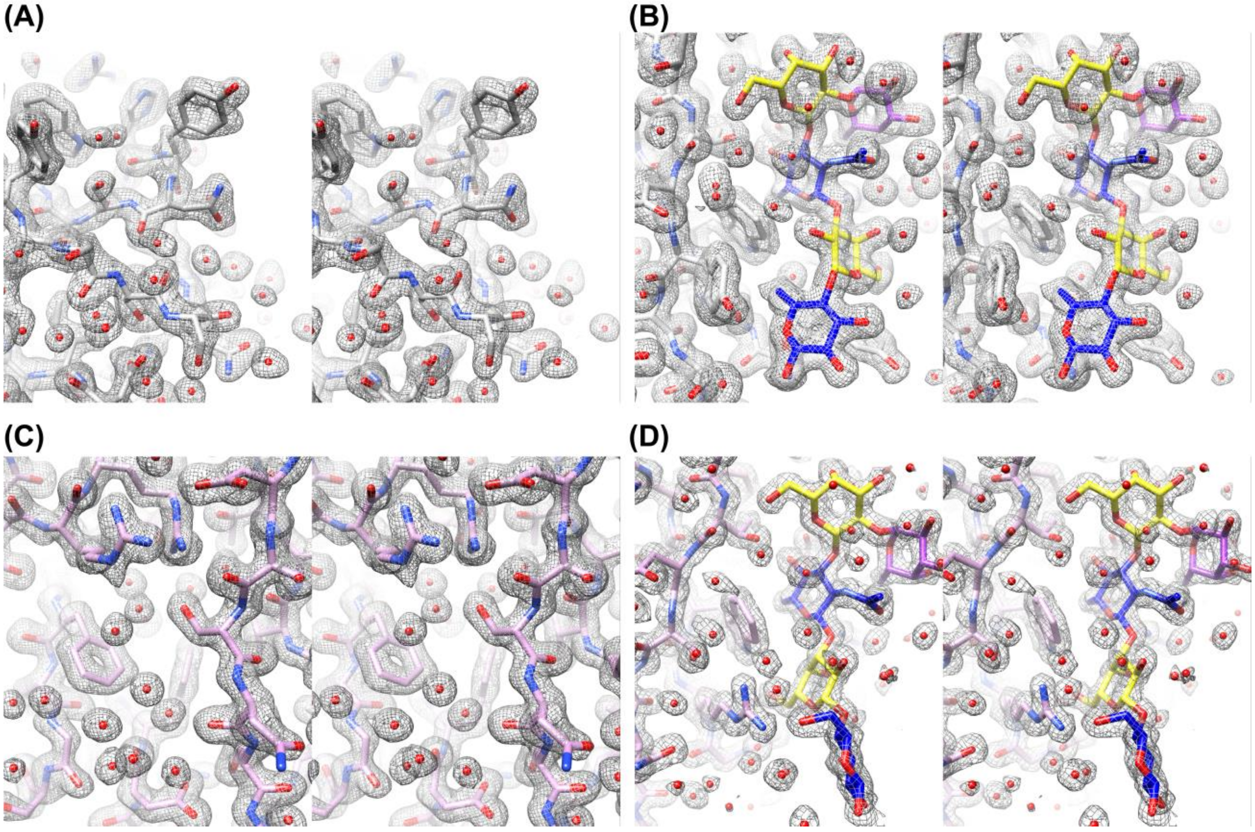

Supplementary Figure 10. Representative electron density maps of the structures reported here. Representative stereo image of 2Fo-Fc maps contoured at 1.0σ (grey mesh) in the structures of P[6] RV3 VP8*, (A) apo and (B) in complex with LNFP1, and the structures of P[4] Indian VP8*, (C) apo and (D) in complex with LNFP1.

Supplementary References

1. Tamura, K., Stecher, G., Peterson, D., Filipski, A. & Kumar, S. MEGA6: Molecular Evolutionary Genetics Analysis version 6.0. *Mol Biol Evol* **30**, 2725-2729, doi:10.1093/molbev/mst197 (2013).
2. Winn, M. D. *et al.* Overview of the CCP4 suite and current developments. *Acta Crystallogr D Biol Crystallogr* **67**, 235-242, doi:S0907444910045749 [pii] 10.1107/S0907444910045749 (2011).
3. Laskowski, R. A. & Swindells, M. B. LigPlot+: multiple ligand-protein interaction diagrams for drug discovery. *J Chem Inf Model* **51**, 2778-2786, doi:10.1021/ci200227u (2011).
